# Authors' Reply

**DOI:** 10.1371/journal.pmed.0030207

**Published:** 2006-04-25

**Authors:** Peter J Hotez, Albert B Sabin, David H Molyneux, Alan Fenwick, Eric Ottesen, Sonia Ehrlich Sachs, Jeffrey D Sachs

**Affiliations:** **1**The George Washington UniversityWashington, D. CUnited States of America; **2**Vaccine InstituteWashington, D. CUnited States of America; **3**Liverpool School of Tropical MedicineLiverpoolUnited Kingdom; **4**Imperial College LondonLondonUnited Kingdom; **5**Schistosomiasis Control InitiativeLondonUnited Kingdom; **6**Emory UniversityAtlanta, GeorgiaUnited States of America; **7**Lymphatic Filariasis Support CenterAtlanta, GeorgiaUnited States of America; **8**Columbia UniversityNew York, New YorkUnited States of America

Damian Walker and Godfrey Walker [ 1] make a strong case for adding congenital syphilis to our proposed list of neglected tropical diseases (NTDs). Indeed, we would hasten to add all of the major treponemal infections—including yaws, endemic syphilis (bejel), and pinta—could potentially qualify as NTDs. Leptospirosis and bartonellosis might also qualify as important neglected bacterial infections, while amoebiasis is an important yet neglected protozoan infection. Therefore, it is possible that in the future our list of 13–15 NTDs could expand to approximately 20 conditions. In the meantime, we are working to establish a set of consensus guidelines for this important list of NTDs.
